# Bis[(2-meth­oxy­phen­yl)di­phenyl­phosphane-κ*P*](nitrito-κ^2^*O*,*O*′)silver(I)

**DOI:** 10.1107/S2414314625001932

**Published:** 2025-03-27

**Authors:** Frederick P. Malan, Kariska Potgieter, Reinout Meijboom

**Affiliations:** a Department of Chemistry, University of Pretoria, Lynnwood Road, Hatfield, Pretoria, 0002, South Africa; b Department of Chemical Sciences, University of Johannesburg, PO Box 524, Auckland Park, 2006, Johannesburg, South Africa

**Keywords:** silver(I) complex, diphenyl-2-meth­oxy­phenyl phosphine, nitrite, crystal structure

## Abstract

The mol­ecular structure of the title Ag^I^ complex, [Ag(NO_2_)(C_19_H_17_OP)_2_], is described, where a distorted tetra­hedral coordination environment for the Ag^I^ atom is apparent within a O_2_P_2_ donor set as the nitrito anion coordinates in a bidentate mode. A fairly large angle for P—Ag—P [129.126 (16)°] is noted. The O—Ag—O chelate angle is = 50.38 (6)° and the P—Ag—O angles lie in the range 99.51 (5) to 118.45 (6)°. In the crystal, C—H⋯O inter­actions are evident.

## Abstract 2

The synthesis and single-crystal structure description of a silver(I) diphenyl-2-meth­oxy­phenyl­phosphine nitrite complex is described.

## Graphical abstract

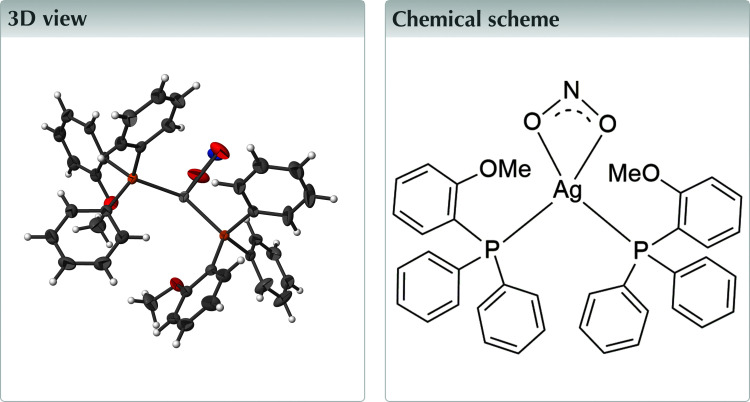

## Structure description

Phosphane-containing silver(I) complexes remain an important class of compounds studied mainly for their potent anti­microbial, anti­bacterial and anti­cancer activity (Potgieter *et al.*, 2016 ▸). To this end, studies of their mol­ecular structures by means of single-crystal X-ray diffraction remain important (Malan *et al.*, 2022 ▸) in order to establish possible structure–activity relationships.

**Figure 1:**
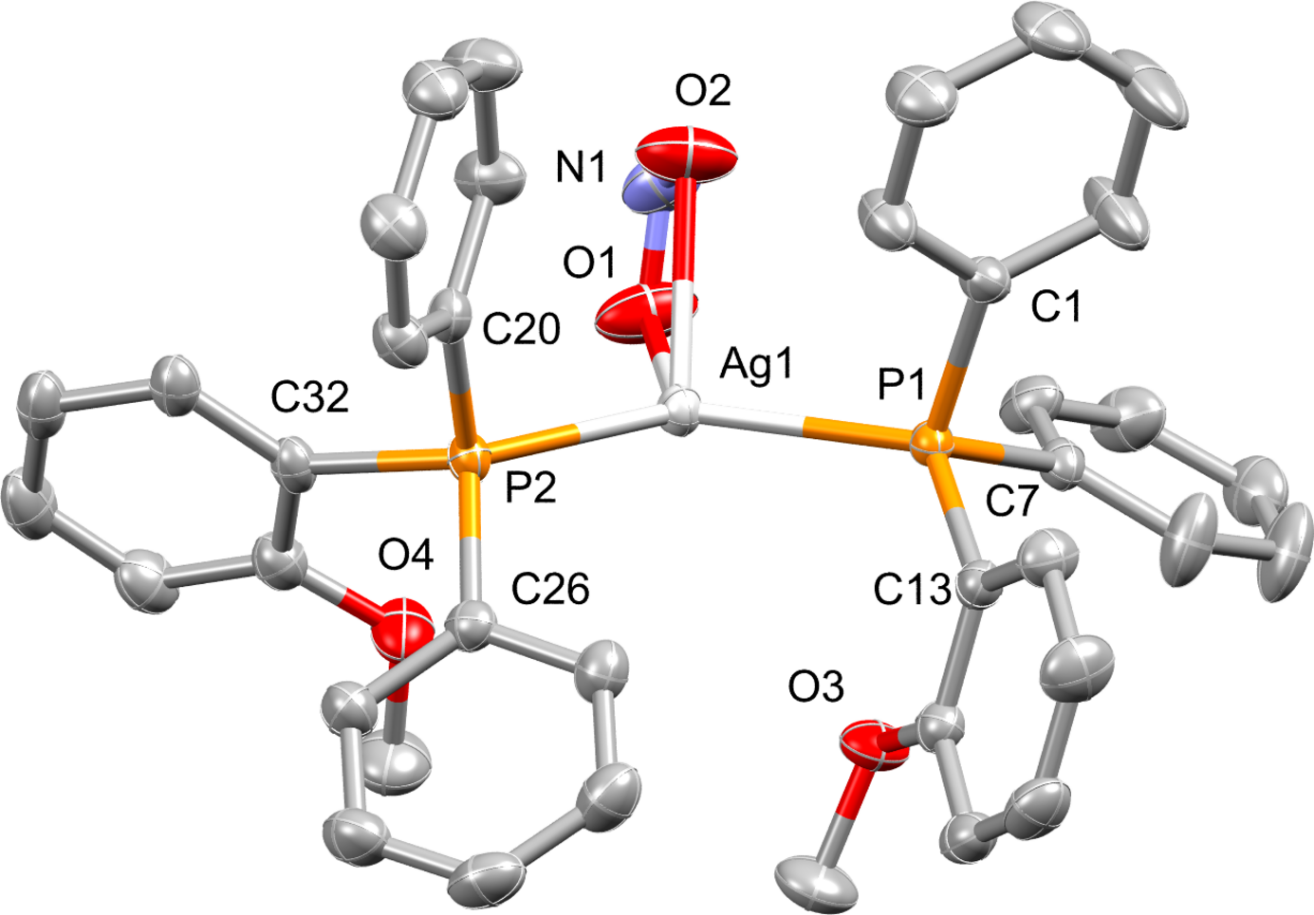
The mol­ecular structure of the title compound showing the atom-labelling scheme and displacement ellipsoids at the 50% probability level. Hydrogen atoms are omitted for clarity.

Fig. 1 ▸ shows the mol­ecular structure of the title compound and Table 1 ▸ lists key geometric parameters. The Ag^I^ complex crystallizes in the monoclinic space group *C*2/*c*, *Z* = 8 with one complete mol­ecule featuring in the asymmetric unit. The distorted tetra­hedral geometry exhibited by the central silver cation comprises a bidentate nitrito ligand, which forms an acute chelate angle of O1—Ag1—O2 = 50.38 (6)°, and two diphen­yl(2-meth­oxy­phen­yl)phosphane ligands, which subtend a wide angle, *i.e.* P1—Ag1—P2 = 129.126 (16)°. The *ipso*-aryl carbon atoms of each of the phosphine ligands overlap in a near-eclipsed fashion when viewed along the P1–Ag1–P2 plane, as indicated by the C1—P1—P2—C20 and C7—P1—P2—C32 torsion angles of −9.50 (8) and −11.27 (14)°, respectively. The C_7_H_7_O aryl groups from each phosphane ligand are adjacent with the oxygen atoms of the OMe groups facing one another, but do not overlap when viewed down the P1–Ag1–P2 plane. The plane defined by atoms P1, Ag1 and P2 inter­cepts the plane defined by the Ag1, O1 and O2 atoms in a near-perpendicular fashion at an angle of 84.13 (6)°. All other bond lengths and angles correlate well with related compounds (Potgieter *et al.*, 2016 ▸).

**Figure 2:**
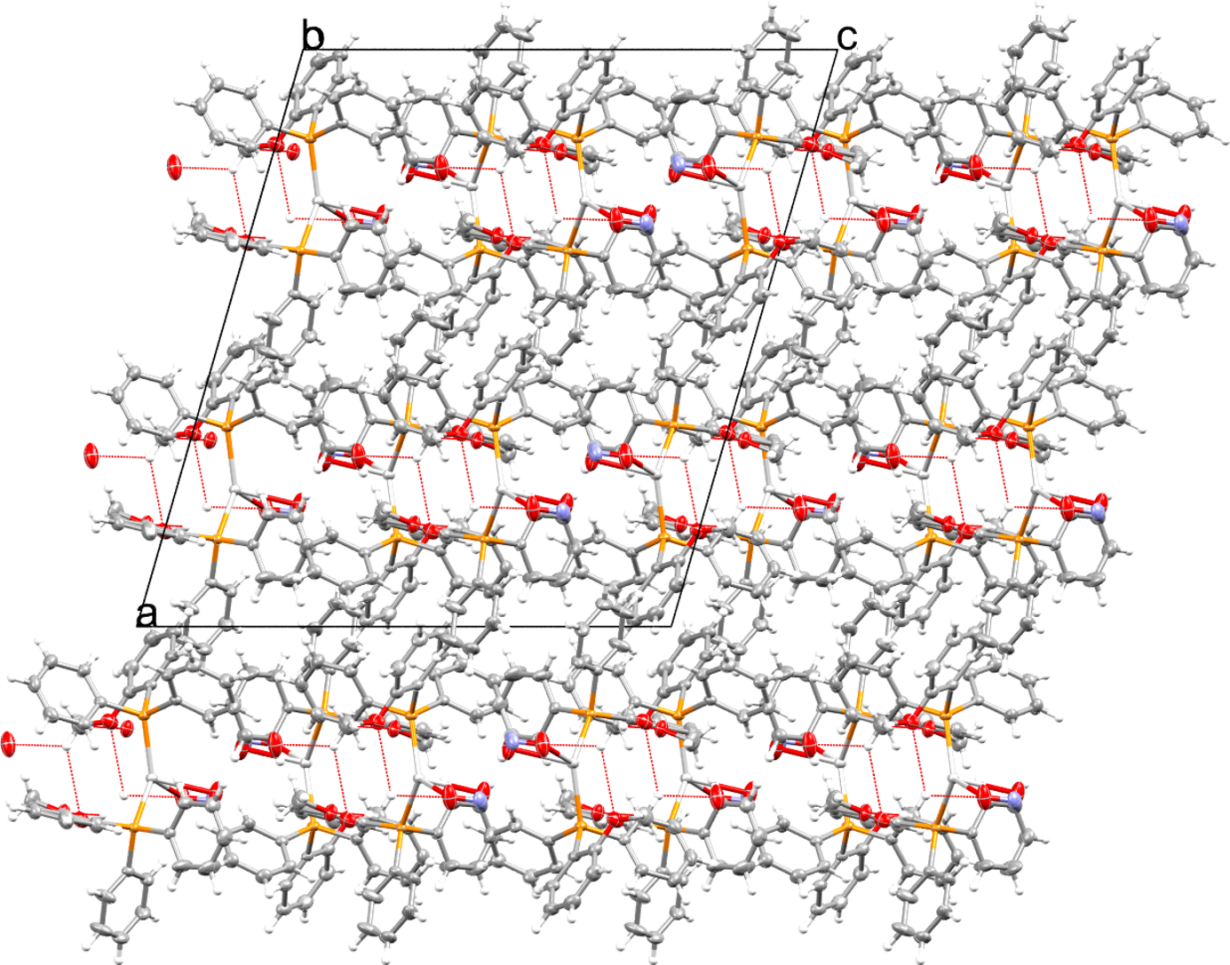
Packing diagram viewed in perspective along the *b* axis. Hydrogen-bonding inter­actions are indicated by means of red dotted lines.

In the crystal, individual complexes pack in three-dimensions as layers of isolated complexes connected *via* weak C—H⋯O hydrogen-bonding inter­actions, Table 2 ▸. These layers pack as alternating phenyl- and oxygen-rich layers, creating alternating hydro­phobic and hydro­philic environments, respectively. A view of the packing with the observed C—H⋯O inter­actions is shown in Fig. 2 ▸.

## Synthesis and crystallization

A 1 mmol solution of silver nitrite was prepared in aceto­nitrile (10 ml) and added to a solution of diphenyl-2-meth­oxy­phenyl­phosphine (2 mmol) in aceto­nitrile (10 ml). The solution was stirred at 80°C, removed from the heat and left to slowly cool and crystallize.

## Refinement

For full experimental details including crystal data, data collection and structure refinement details, refer to Table 3 ▸. The maximum and minimum residual electron density peaks are located 0.86 and 0.61 Å, respectively, from the Ag1 atom

## Supplementary Material

Crystal structure: contains datablock(s) I. DOI: 10.1107/S2414314625001932/tk4116sup1.cif

Structure factors: contains datablock(s) I. DOI: 10.1107/S2414314625001932/tk4116Isup2.hkl

Supporting information file. DOI: 10.1107/S2414314625001932/tk4116Isup3.cdx

CCDC reference: 2427754

Additional supporting information:  crystallographic information; 3D view; checkCIF report

## Figures and Tables

**Table 1:** Selected geometric parameters (Å, °)

Ag1—O1	2.3931 (16)	Ag1—P1	2.4283 (4)
Ag1—O2	2.4927 (17)	Ag1—P2	2.4136 (4)
			
O1—Ag1—P1	112.41 (6)	O2—Ag1—P1	99.51 (5)
O1—Ag1—P2	118.45 (6)	O2—Ag1—P2	111.52 (5)
O1—Ag1—O2	50.38 (6)	P1—Ag1—P2	129.126 (16)

**Table 2:** Hydrogen-bond geometry (Å, °)

*D*—H⋯*A*	*D*—H	H⋯*A*	*D*⋯*A*	*D*—H⋯*A*
N2—H1N⋯O2^i^	0.87 (1)	2.00 (1)	2.8634 (18)	173 (2)
C17—H17⋯O2^ii^	0.95	2.37	3.225 (3)	149
C38—H38*C*⋯O1^iii^	0.98	2.45	2.936 (3)	110

Symmetry codes: (i) 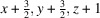; (ii) 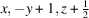; (iii) 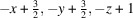.

**Table 3:** Experimental details

Crystal data	
Chemical formula	[Ag(NO_2_)(C_19_H_17_OP)_2_]
*M*_r_	738.47
Crystal system, space group	Monoclinic, *C*2/*c*
Temperature (K)	150
*a*, *b*, *c* (Å)	22.6427 (3), 15.6971 (2), 20.1469 (3)
β (°)	106.080 (2)
*V* (Å^3^)	6880.55 (18)
*Z*	8
Radiation type	Mo *K*α
μ (mm^−1^)	0.72
Crystal size (mm)	0.31 × 0.27 × 0.22

Data collection	
Diffractometer	XtaLAB Synergy R, DW system, HyPix
Absorption correction	Multi-scan (*CrysAlis PRO*; Rigaku OD, 2022 ▸)
*T*_min_, *T*_max_	0.038, 1.000
No. of measured, independent and observed [*I* > 2σ(*I*)] reflections	56728, 9287, 7975
*R*_int_	0.036
(sin θ/λ)_max_ (Å^−1^)	0.727

Refinement	
*R*[*F*^2^ > 2σ(*F*^2^)], *wR*(*F*^2^), *S*	0.031, 0.076, 1.07
No. of reflections	9287
No. of parameters	417
H-atom treatment	H-atom parameters constrained
Δρ_max_, Δρ_min_ (e Å^−3^)	1.69, −0.93

Computer programs: *CrysAlis PRO* (Rigaku OD, 2022 ▸), *SHELXT* (Sheldrick, 2015*a* ▸), *SHELXL* (Sheldrick, 2015*b* ▸) and *OLEX2* (Dolomanov *et al.*, 2009 ▸).

## full crystallographic data

### Bis[(2-methoxyphenyl)diphenylphosphane-κP](nitrito-κ2O,O')silver(I) Crystal data

**Table:**

[Ag(NO_2_)(C_19_H_17_OP)_2_]	*F*(000) = 3016
*M**_r_* = 738.47	*D*_x_ = 1.420 Mg m^−^^3^
Monoclinic, *C*2/*c*	Mo *K*α radiation, λ = 0.71073 Å
*a* = 22.6427 (3) Å	Cell parameters from 36798 reflections
*b* = 15.6971 (2) Å	θ = 2.8–31.3°
*c* = 20.1469 (3) Å	µ = 0.72 mm^−^^1^
β = 106.080 (2)°	*T* = 150 K
*V* = 6880.55 (18) Å^3^	Block, colourless
*Z* = 8	0.31 × 0.27 × 0.22 mm

### Bis[(2-methoxyphenyl)diphenylphosphane-κP](nitrito-κ2O,O')silver(I) Data collection

**Table:**

XtaLAB Synergy R, DW system, HyPix diffractometer	9287 independent reflections
Radiation source: Rotating-anode X-ray tube, Rigaku (Mo) X-ray Source	7975 reflections with *I* > 2σ(*I*)
Mirror monochromator	*R*_int_ = 0.036
Detector resolution: 10.0000 pixels mm^-1^	θ_max_ = 31.1°, θ_min_ = 2.6°
ω scans	*h* = −32→31
Absorption correction: multi-scan (CrysAlisPro; Rigaku OD, 2022)	*k* = −19→21
*T*_min_ = 0.038, *T*_max_ = 1.000	*l* = −28→26
56728 measured reflections	

### Bis[(2-methoxyphenyl)diphenylphosphane-κP](nitrito-κ2O,O')silver(I) Refinement

**Table:**

Refinement on *F*^2^	Primary atom site location: dual
Least-squares matrix: full	Hydrogen site location: inferred from neighbouring sites
*R*[*F*^2^ > 2σ(*F*^2^)] = 0.031	H-atom parameters constrained
*wR*(*F*^2^) = 0.076	*w* = 1/[σ^2^(*F*_o_^2^) + (0.0351*P*)^2^ + 8.2387*P*] where *P* = (*F*_o_^2^ + 2*F*_c_^2^)/3
*S* = 1.07	(Δ/σ)_max_ = 0.003
9287 reflections	Δρ_max_ = 1.69 e Å^−^^3^
417 parameters	Δρ_min_ = −0.93 e Å^−^^3^
0 restraints	

### Bis[(2-methoxyphenyl)diphenylphosphane-κP](nitrito-κ2O,O')silver(I) Special details

**Table:**

Geometry. All esds (except the esd in the dihedral angle between two l.s. planes) are estimated using the full covariance matrix. The cell esds are taken into account individually in the estimation of esds in distances, angles and torsion angles; correlations between esds in cell parameters are only used when they are defined by crystal symmetry. An approximate (isotropic) treatment of cell esds is used for estimating esds involving l.s. planes.

### Bis[(2-methoxyphenyl)diphenylphosphane-κP](nitrito-κ2O,O')silver(I) Fractional atomic coordinates and isotropic or equivalent isotropic displacement parameters (Å^2^)

**Table:**

	*x*	*y*	*z*	*U*_iso_*/*U*_eq_	
Ag1	0.73700 (2)	0.56462 (2)	0.38969 (2)	0.02206 (5)	
P1	0.65137 (2)	0.47508 (3)	0.39633 (2)	0.01960 (9)	
P2	0.84579 (2)	0.54168 (3)	0.43873 (2)	0.02195 (9)	
O3	0.67728 (7)	0.53424 (9)	0.53680 (7)	0.0338 (3)	
O4	0.83502 (7)	0.69983 (10)	0.49956 (8)	0.0400 (3)	
O2	0.71068 (10)	0.58520 (12)	0.26215 (9)	0.0567 (5)	
O1	0.70419 (10)	0.69047 (11)	0.32257 (9)	0.0568 (5)	
C18	0.67471 (8)	0.44718 (11)	0.53731 (10)	0.0251 (4)	
C13	0.66033 (8)	0.40809 (11)	0.47215 (9)	0.0230 (3)	
C26	0.86925 (8)	0.49997 (11)	0.52714 (9)	0.0244 (3)	
C1	0.64164 (8)	0.39815 (11)	0.32640 (9)	0.0238 (3)	
C20	0.87211 (8)	0.46280 (11)	0.38685 (9)	0.0227 (3)	
C17	0.68459 (9)	0.39840 (14)	0.59698 (10)	0.0322 (4)	
H17	0.6944	0.4252	0.6410	0.039*	
N1	0.69803 (10)	0.66201 (13)	0.26371 (10)	0.0453 (5)	
C32	0.89811 (8)	0.63093 (11)	0.44337 (9)	0.0239 (3)	
C7	0.57573 (8)	0.52308 (11)	0.38389 (9)	0.0236 (3)	
C16	0.68000 (10)	0.31062 (14)	0.59185 (12)	0.0380 (5)	
H16	0.6867	0.2773	0.6326	0.046*	
C27	0.83098 (9)	0.44130 (12)	0.54679 (11)	0.0318 (4)	
H27	0.7936	0.4244	0.5146	0.038*	
C34	0.98944 (9)	0.69799 (13)	0.42789 (11)	0.0338 (4)	
H34	1.0243	0.6965	0.4104	0.041*	
C14	0.65625 (10)	0.31942 (12)	0.46864 (11)	0.0321 (4)	
H14	0.6468	0.2920	0.4249	0.038*	
C25	0.91678 (8)	0.40200 (12)	0.41489 (10)	0.0279 (4)	
H25	0.9363	0.4017	0.4631	0.033*	
C35	0.97823 (10)	0.76861 (13)	0.46359 (12)	0.0393 (5)	
H35	1.0062	0.8151	0.4713	0.047*	
C33	0.94915 (8)	0.62915 (12)	0.41780 (10)	0.0275 (4)	
H33	0.9566	0.5805	0.3932	0.033*	
C28	0.84699 (11)	0.40731 (15)	0.61304 (12)	0.0413 (5)	
H28	0.8210	0.3665	0.6256	0.050*	
C23	0.90500 (10)	0.34138 (14)	0.30238 (11)	0.0364 (4)	
H23	0.9159	0.2996	0.2737	0.044*	
C37	0.88670 (9)	0.70369 (12)	0.47768 (10)	0.0295 (4)	
C36	0.92706 (10)	0.77295 (13)	0.48830 (11)	0.0366 (5)	
H36	0.9195	0.8222	0.5121	0.044*	
C29	0.90059 (11)	0.43267 (15)	0.66074 (12)	0.0420 (5)	
H29	0.9114	0.4095	0.7061	0.050*	
C21	0.84448 (10)	0.46245 (14)	0.31582 (11)	0.0358 (4)	
H21	0.8140	0.5037	0.2959	0.043*	
C24	0.93294 (10)	0.34175 (13)	0.37256 (11)	0.0344 (4)	
H24	0.9635	0.3004	0.3921	0.041*	
C2	0.69423 (9)	0.36501 (14)	0.31357 (11)	0.0357 (5)	
H2	0.7334	0.3835	0.3407	0.043*	
C31	0.92311 (9)	0.52493 (13)	0.57568 (11)	0.0329 (4)	
H31	0.9496	0.5649	0.5631	0.039*	
C10	0.46111 (10)	0.60076 (16)	0.36229 (12)	0.0425 (5)	
H10	0.4217	0.6265	0.3539	0.051*	
C15	0.66583 (11)	0.27086 (14)	0.52812 (12)	0.0417 (5)	
H15	0.6627	0.2105	0.5251	0.050*	
C4	0.63425 (12)	0.27957 (15)	0.22160 (12)	0.0452 (6)	
H4	0.6315	0.2396	0.1854	0.054*	
C19	0.69918 (13)	0.57647 (16)	0.60203 (12)	0.0484 (6)	
H19A	0.7034	0.6376	0.5944	0.073*	
H19B	0.7392	0.5529	0.6271	0.073*	
H19C	0.6699	0.5679	0.6292	0.073*	
C30	0.93856 (10)	0.49199 (15)	0.64233 (11)	0.0402 (5)	
H30	0.9751	0.5101	0.6752	0.048*	
C11	0.50465 (10)	0.63502 (16)	0.33463 (12)	0.0420 (5)	
H11	0.4957	0.6859	0.3083	0.050*	
C3	0.69051 (11)	0.30542 (15)	0.26173 (12)	0.0406 (5)	
H3	0.7270	0.2826	0.2541	0.049*	
C8	0.53234 (10)	0.49021 (15)	0.41353 (15)	0.0477 (6)	
H8	0.5417	0.4408	0.4418	0.057*	
C22	0.86113 (11)	0.40218 (16)	0.27396 (11)	0.0428 (5)	
H22	0.8423	0.4027	0.2256	0.051*	
C12	0.56175 (9)	0.59634 (14)	0.34460 (11)	0.0332 (4)	
H12	0.5912	0.6203	0.3244	0.040*	
C38	0.82967 (13)	0.75616 (16)	0.55317 (14)	0.0510 (6)	
H38A	0.8268	0.8150	0.5363	0.076*	
H38B	0.8659	0.7502	0.5930	0.076*	
H38C	0.7926	0.7419	0.5670	0.076*	
C9	0.47504 (11)	0.52905 (17)	0.40224 (17)	0.0562 (7)	
H9	0.4454	0.5056	0.4224	0.067*	
C6	0.58518 (10)	0.37091 (18)	0.28587 (14)	0.0539 (7)	
H6	0.5485	0.3928	0.2936	0.065*	
C5	0.58173 (12)	0.3116 (2)	0.23378 (16)	0.0690 (10)	
H5	0.5427	0.2931	0.2063	0.083*	

### Bis[(2-methoxyphenyl)diphenylphosphane-κP](nitrito-κ2O,O')silver(I) Atomic displacement parameters (Å^2^)

**Table:**

	*U*^11^	*U*^22^	*U*^33^	*U*^12^	*U*^13^	*U*^23^
Ag1	0.01771 (7)	0.02421 (7)	0.02355 (7)	−0.00003 (4)	0.00456 (5)	0.00047 (5)
P1	0.01714 (19)	0.0214 (2)	0.0201 (2)	−0.00046 (15)	0.00497 (16)	−0.00116 (15)
P2	0.01636 (19)	0.0242 (2)	0.0244 (2)	−0.00045 (15)	0.00414 (16)	−0.00035 (16)
O3	0.0460 (8)	0.0288 (7)	0.0254 (7)	−0.0012 (6)	0.0078 (6)	−0.0070 (5)
O4	0.0390 (8)	0.0365 (8)	0.0483 (9)	−0.0034 (6)	0.0184 (7)	−0.0163 (7)
O2	0.0876 (15)	0.0510 (10)	0.0287 (8)	0.0137 (10)	0.0113 (9)	0.0040 (7)
O1	0.1013 (15)	0.0368 (9)	0.0415 (10)	0.0125 (9)	0.0353 (10)	0.0088 (7)
C18	0.0219 (8)	0.0288 (9)	0.0257 (9)	0.0007 (6)	0.0085 (7)	−0.0002 (7)
C13	0.0210 (8)	0.0252 (8)	0.0236 (8)	−0.0008 (6)	0.0074 (6)	0.0009 (7)
C26	0.0220 (8)	0.0271 (9)	0.0246 (9)	0.0034 (6)	0.0073 (7)	0.0000 (7)
C1	0.0237 (8)	0.0262 (9)	0.0204 (8)	0.0004 (6)	0.0041 (7)	−0.0016 (6)
C20	0.0174 (7)	0.0239 (8)	0.0270 (9)	−0.0015 (6)	0.0064 (6)	0.0006 (7)
C17	0.0273 (9)	0.0458 (11)	0.0249 (9)	0.0030 (8)	0.0096 (7)	0.0026 (8)
N1	0.0539 (12)	0.0497 (12)	0.0360 (11)	0.0107 (9)	0.0188 (9)	0.0153 (9)
C32	0.0195 (8)	0.0236 (8)	0.0255 (9)	−0.0009 (6)	0.0011 (7)	0.0042 (7)
C7	0.0181 (8)	0.0262 (9)	0.0262 (9)	−0.0001 (6)	0.0060 (7)	−0.0046 (7)
C16	0.0393 (11)	0.0434 (12)	0.0343 (11)	0.0055 (9)	0.0150 (9)	0.0152 (9)
C27	0.0268 (9)	0.0325 (10)	0.0371 (11)	0.0003 (7)	0.0108 (8)	0.0023 (8)
C34	0.0252 (9)	0.0364 (10)	0.0376 (11)	−0.0045 (7)	0.0050 (8)	0.0125 (8)
C14	0.0405 (11)	0.0254 (9)	0.0323 (10)	−0.0024 (8)	0.0135 (8)	−0.0001 (7)
C25	0.0269 (9)	0.0254 (9)	0.0283 (9)	0.0014 (7)	0.0024 (7)	0.0015 (7)
C35	0.0402 (11)	0.0315 (10)	0.0409 (12)	−0.0139 (8)	0.0022 (9)	0.0082 (9)
C33	0.0216 (8)	0.0277 (9)	0.0307 (10)	0.0006 (6)	0.0031 (7)	0.0074 (7)
C28	0.0398 (12)	0.0451 (12)	0.0438 (13)	0.0035 (9)	0.0195 (10)	0.0119 (10)
C23	0.0358 (11)	0.0361 (11)	0.0375 (11)	0.0034 (8)	0.0107 (9)	−0.0086 (9)
C37	0.0285 (9)	0.0288 (9)	0.0287 (10)	−0.0007 (7)	0.0036 (7)	0.0008 (7)
C36	0.0446 (12)	0.0258 (9)	0.0360 (11)	−0.0058 (8)	0.0054 (9)	−0.0010 (8)
C29	0.0481 (13)	0.0514 (13)	0.0292 (11)	0.0116 (10)	0.0155 (10)	0.0096 (9)
C21	0.0320 (10)	0.0457 (12)	0.0278 (10)	0.0148 (9)	0.0051 (8)	0.0016 (8)
C24	0.0341 (10)	0.0271 (9)	0.0390 (11)	0.0073 (8)	0.0051 (8)	−0.0005 (8)
C2	0.0251 (9)	0.0408 (11)	0.0384 (11)	0.0032 (8)	0.0044 (8)	−0.0145 (9)
C31	0.0290 (10)	0.0385 (11)	0.0292 (10)	−0.0022 (8)	0.0047 (8)	0.0011 (8)
C10	0.0251 (10)	0.0562 (14)	0.0463 (13)	0.0119 (9)	0.0101 (9)	−0.0068 (11)
C15	0.0572 (14)	0.0280 (10)	0.0435 (13)	0.0013 (9)	0.0200 (11)	0.0098 (9)
C4	0.0517 (14)	0.0453 (13)	0.0349 (12)	0.0017 (10)	0.0059 (10)	−0.0187 (10)
C19	0.0617 (16)	0.0452 (13)	0.0339 (12)	−0.0027 (11)	0.0061 (11)	−0.0173 (10)
C30	0.0372 (11)	0.0517 (13)	0.0279 (10)	0.0031 (9)	0.0027 (9)	−0.0009 (9)
C11	0.0353 (11)	0.0538 (13)	0.0372 (12)	0.0201 (10)	0.0105 (9)	0.0092 (10)
C3	0.0404 (11)	0.0422 (12)	0.0401 (12)	0.0078 (9)	0.0129 (10)	−0.0113 (9)
C8	0.0327 (11)	0.0376 (12)	0.0807 (19)	0.0031 (9)	0.0288 (12)	0.0167 (11)
C22	0.0419 (12)	0.0582 (14)	0.0259 (10)	0.0143 (10)	0.0053 (9)	−0.0039 (9)
C12	0.0278 (9)	0.0426 (11)	0.0313 (10)	0.0093 (8)	0.0117 (8)	0.0064 (8)
C38	0.0599 (16)	0.0471 (14)	0.0502 (15)	−0.0016 (11)	0.0223 (12)	−0.0179 (11)
C9	0.0295 (11)	0.0558 (15)	0.093 (2)	0.0031 (10)	0.0330 (13)	0.0108 (14)
C6	0.0234 (10)	0.0749 (17)	0.0563 (15)	0.0024 (10)	−0.0006 (10)	−0.0378 (13)
C5	0.0369 (13)	0.092 (2)	0.0661 (18)	0.0001 (13)	−0.0062 (12)	−0.0520 (17)

### Bis[(2-methoxyphenyl)diphenylphosphane-κP](nitrito-κ2O,O')silver(I) Geometric parameters (Å, º)

**Table:**

Ag1—O1	2.3931 (16)	C35—C36	1.384 (3)
Ag1—O2	2.4927 (17)	C33—H33	0.9500
Ag1—P1	2.4283 (4)	C28—H28	0.9500
Ag1—P2	2.4136 (4)	C28—C29	1.382 (3)
P1—C13	1.8190 (18)	C23—H23	0.9500
P1—C1	1.8220 (18)	C23—C24	1.380 (3)
P1—C7	1.8238 (17)	C23—C22	1.382 (3)
P2—C26	1.8328 (19)	C37—C36	1.398 (3)
P2—C20	1.8237 (18)	C36—H36	0.9500
P2—C32	1.8204 (18)	C29—H29	0.9500
O3—C18	1.368 (2)	C29—C30	1.386 (3)
O3—C19	1.432 (2)	C21—H21	0.9500
O4—C37	1.361 (2)	C21—C22	1.387 (3)
O4—C38	1.427 (3)	C24—H24	0.9500
O2—N1	1.242 (3)	C2—H2	0.9500
O1—N1	1.238 (3)	C2—C3	1.387 (3)
C18—C13	1.403 (3)	C31—H31	0.9500
C18—C17	1.390 (3)	C31—C30	1.390 (3)
C13—C14	1.395 (3)	C10—H10	0.9500
C26—C27	1.395 (3)	C10—C11	1.370 (3)
C26—C31	1.392 (3)	C10—C9	1.369 (4)
C1—C2	1.388 (3)	C15—H15	0.9500
C1—C6	1.380 (3)	C4—H4	0.9500
C20—C25	1.392 (2)	C4—C3	1.368 (3)
C20—C21	1.394 (3)	C4—C5	1.375 (4)
C17—H17	0.9500	C19—H19A	0.9800
C17—C16	1.383 (3)	C19—H19B	0.9800
C32—C33	1.390 (3)	C19—H19C	0.9800
C32—C37	1.396 (3)	C30—H30	0.9500
C7—C8	1.383 (3)	C11—H11	0.9500
C7—C12	1.382 (3)	C11—C12	1.392 (3)
C16—H16	0.9500	C3—H3	0.9500
C16—C15	1.383 (3)	C8—H8	0.9500
C27—H27	0.9500	C8—C9	1.394 (3)
C27—C28	1.389 (3)	C22—H22	0.9500
C34—H34	0.9500	C12—H12	0.9500
C34—C35	1.383 (3)	C38—H38A	0.9800
C34—C33	1.392 (3)	C38—H38B	0.9800
C14—H14	0.9500	C38—H38C	0.9800
C14—C15	1.386 (3)	C9—H9	0.9500
C25—H25	0.9500	C6—H6	0.9500
C25—C24	1.389 (3)	C6—C5	1.388 (3)
C35—H35	0.9500	C5—H5	0.9500
			
O1—Ag1—P1	112.41 (6)	C24—C23—C22	119.64 (19)
O1—Ag1—P2	118.45 (6)	C22—C23—H23	120.2
O1—Ag1—O2	50.38 (6)	O4—C37—C32	114.68 (16)
O2—Ag1—P1	99.51 (5)	O4—C37—C36	124.68 (19)
O2—Ag1—P2	111.52 (5)	C32—C37—C36	120.63 (19)
P1—Ag1—P2	129.126 (16)	C35—C36—C37	118.9 (2)
C13—P1—Ag1	118.42 (6)	C35—C36—H36	120.5
C13—P1—C1	103.16 (8)	C37—C36—H36	120.5
C13—P1—C7	103.67 (8)	C28—C29—H29	120.1
C1—P1—Ag1	105.57 (6)	C28—C29—C30	119.9 (2)
C1—P1—C7	105.05 (8)	C30—C29—H29	120.1
C7—P1—Ag1	119.15 (6)	C20—C21—H21	119.8
C26—P2—Ag1	116.06 (6)	C22—C21—C20	120.43 (19)
C20—P2—Ag1	108.97 (6)	C22—C21—H21	119.8
C20—P2—C26	105.17 (8)	C25—C24—H24	119.7
C32—P2—Ag1	118.82 (6)	C23—C24—C25	120.53 (18)
C32—P2—C26	102.46 (8)	C23—C24—H24	119.7
C32—P2—C20	103.93 (8)	C1—C2—H2	119.4
C18—O3—C19	117.40 (17)	C3—C2—C1	121.11 (19)
C37—O4—C38	118.59 (18)	C3—C2—H2	119.4
N1—O2—Ag1	95.21 (13)	C26—C31—H31	119.6
N1—O1—Ag1	100.27 (13)	C30—C31—C26	120.74 (19)
O3—C18—C13	115.42 (16)	C30—C31—H31	119.6
O3—C18—C17	124.04 (17)	C11—C10—H10	120.4
C17—C18—C13	120.54 (17)	C9—C10—H10	120.4
C18—C13—P1	118.41 (13)	C9—C10—C11	119.17 (19)
C14—C13—P1	122.93 (14)	C16—C15—C14	119.7 (2)
C14—C13—C18	118.60 (17)	C16—C15—H15	120.2
C27—C26—P2	118.43 (14)	C14—C15—H15	120.2
C31—C26—P2	122.95 (15)	C3—C4—H4	120.1
C31—C26—C27	118.61 (18)	C3—C4—C5	119.7 (2)
C2—C1—P1	117.79 (14)	C5—C4—H4	120.1
C6—C1—P1	123.82 (15)	O3—C19—H19A	109.5
C6—C1—C2	118.40 (18)	O3—C19—H19B	109.5
C25—C20—P2	123.31 (14)	O3—C19—H19C	109.5
C25—C20—C21	118.88 (17)	H19A—C19—H19B	109.5
C21—C20—P2	117.78 (14)	H19A—C19—H19C	109.5
C18—C17—H17	120.2	H19B—C19—H19C	109.5
C16—C17—C18	119.55 (19)	C29—C30—C31	120.0 (2)
C16—C17—H17	120.2	C29—C30—H30	120.0
O1—N1—O2	114.14 (18)	C31—C30—H30	120.0
C33—C32—P2	124.17 (14)	C10—C11—H11	119.5
C33—C32—C37	119.15 (17)	C10—C11—C12	121.0 (2)
C37—C32—P2	116.63 (14)	C12—C11—H11	119.5
C8—C7—P1	122.60 (15)	C2—C3—H3	120.1
C12—C7—P1	118.68 (14)	C4—C3—C2	119.8 (2)
C12—C7—C8	118.70 (17)	C4—C3—H3	120.1
C17—C16—H16	119.6	C7—C8—H8	119.8
C15—C16—C17	120.81 (19)	C7—C8—C9	120.4 (2)
C15—C16—H16	119.6	C9—C8—H8	119.8
C26—C27—H27	119.7	C23—C22—C21	120.3 (2)
C28—C27—C26	120.6 (2)	C23—C22—H22	119.9
C28—C27—H27	119.7	C21—C22—H22	119.9
C35—C34—H34	120.3	C7—C12—C11	120.12 (19)
C35—C34—C33	119.40 (19)	C7—C12—H12	119.9
C33—C34—H34	120.3	C11—C12—H12	119.9
C13—C14—H14	119.6	O4—C38—H38A	109.5
C15—C14—C13	120.83 (19)	O4—C38—H38B	109.5
C15—C14—H14	119.6	O4—C38—H38C	109.5
C20—C25—H25	119.9	H38A—C38—H38B	109.5
C24—C25—C20	120.22 (18)	H38A—C38—H38C	109.5
C24—C25—H25	119.9	H38B—C38—H38C	109.5
C34—C35—H35	119.4	C10—C9—C8	120.5 (2)
C34—C35—C36	121.28 (18)	C10—C9—H9	119.7
C36—C35—H35	119.4	C8—C9—H9	119.7
C32—C33—C34	120.58 (19)	C1—C6—H6	119.9
C32—C33—H33	119.7	C1—C6—C5	120.2 (2)
C34—C33—H33	119.7	C5—C6—H6	119.9
C27—C28—H28	119.9	C4—C5—C6	120.7 (2)
C29—C28—C27	120.2 (2)	C4—C5—H5	119.7
C29—C28—H28	119.9	C6—C5—H5	119.7
C24—C23—H23	120.2		
			
Ag1—P1—C13—C18	57.99 (15)	C1—C2—C3—C4	1.1 (4)
Ag1—P1—C13—C14	−119.05 (15)	C1—C6—C5—C4	−0.3 (5)
Ag1—P1—C1—C2	39.46 (17)	C20—P2—C26—C27	−84.40 (16)
Ag1—P1—C1—C6	−140.8 (2)	C20—P2—C26—C31	96.36 (17)
Ag1—P1—C7—C8	−152.19 (17)	C20—P2—C32—C33	−6.09 (17)
Ag1—P1—C7—C12	26.26 (18)	C20—P2—C32—C37	176.78 (14)
Ag1—P2—C26—C27	36.10 (16)	C20—C25—C24—C23	0.0 (3)
Ag1—P2—C26—C31	−143.14 (14)	C20—C21—C22—C23	0.5 (4)
Ag1—P2—C20—C25	−143.42 (14)	C17—C18—C13—P1	−177.25 (14)
Ag1—P2—C20—C21	34.67 (17)	C17—C18—C13—C14	−0.1 (3)
Ag1—P2—C32—C33	−127.34 (14)	C17—C16—C15—C14	0.2 (3)
Ag1—P2—C32—C37	55.53 (16)	C32—P2—C26—C27	167.22 (15)
Ag1—O2—N1—O1	0.4 (2)	C32—P2—C26—C31	−12.02 (18)
Ag1—O1—N1—O2	−0.5 (2)	C32—P2—C20—C25	88.96 (16)
P1—C13—C14—C15	177.41 (17)	C32—P2—C20—C21	−92.95 (16)
P1—C1—C2—C3	179.24 (18)	C32—C37—C36—C35	0.7 (3)
P1—C1—C6—C5	−179.6 (3)	C7—P1—C13—C18	−76.55 (15)
P1—C7—C8—C9	−179.8 (2)	C7—P1—C13—C14	106.40 (16)
P1—C7—C12—C11	−179.40 (17)	C7—P1—C1—C2	166.23 (16)
P2—C26—C27—C28	179.45 (16)	C7—P1—C1—C6	−14.0 (2)
P2—C26—C31—C30	179.38 (16)	C7—C8—C9—C10	−0.7 (5)
P2—C20—C25—C24	177.53 (15)	C27—C26—C31—C30	0.1 (3)
P2—C20—C21—C22	−177.89 (18)	C27—C28—C29—C30	−0.2 (3)
P2—C32—C33—C34	−175.26 (14)	C34—C35—C36—C37	1.0 (3)
P2—C32—C37—O4	−3.9 (2)	C25—C20—C21—C22	0.3 (3)
P2—C32—C37—C36	175.20 (15)	C35—C34—C33—C32	−0.1 (3)
O3—C18—C13—P1	3.5 (2)	C33—C32—C37—O4	178.77 (17)
O3—C18—C13—C14	−179.31 (17)	C33—C32—C37—C36	−2.1 (3)
O3—C18—C17—C16	179.00 (18)	C33—C34—C35—C36	−1.3 (3)
O4—C37—C36—C35	179.74 (19)	C28—C29—C30—C31	−0.9 (3)
C18—C13—C14—C15	0.4 (3)	C37—C32—C33—C34	1.8 (3)
C18—C17—C16—C15	0.1 (3)	C21—C20—C25—C24	−0.5 (3)
C13—P1—C1—C2	−85.46 (17)	C24—C23—C22—C21	−1.1 (4)
C13—P1—C1—C6	94.3 (2)	C2—C1—C6—C5	0.1 (4)
C13—P1—C7—C8	−18.1 (2)	C31—C26—C27—C28	−1.3 (3)
C13—P1—C7—C12	160.40 (16)	C10—C11—C12—C7	−1.2 (4)
C13—C18—C17—C16	−0.2 (3)	C19—O3—C18—C13	−172.44 (18)
C13—C14—C15—C16	−0.4 (3)	C19—O3—C18—C17	8.4 (3)
C26—P2—C20—C25	−18.35 (17)	C11—C10—C9—C8	−1.3 (4)
C26—P2—C20—C21	159.74 (16)	C3—C4—C5—C6	0.9 (5)
C26—P2—C32—C33	103.24 (16)	C8—C7—C12—C11	−0.9 (3)
C26—P2—C32—C37	−73.90 (15)	C22—C23—C24—C25	0.8 (3)
C26—C27—C28—C29	1.3 (3)	C12—C7—C8—C9	1.8 (4)
C26—C31—C30—C29	1.0 (3)	C38—O4—C37—C32	159.7 (2)
C1—P1—C13—C18	174.09 (14)	C38—O4—C37—C36	−19.4 (3)
C1—P1—C13—C14	−2.95 (18)	C9—C10—C11—C12	2.3 (4)
C1—P1—C7—C8	89.9 (2)	C6—C1—C2—C3	−0.5 (3)
C1—P1—C7—C12	−91.66 (17)	C5—C4—C3—C2	−1.2 (4)

### Bis[(2-methoxyphenyl)diphenylphosphane-κP](nitrito-κ2O,O')silver(I) Hydrogen-bond geometry (Å, º)

**Table:**

*D*—H···*A*	*D*—H	H···*A*	*D*···*A*	*D*—H···*A*
N2—H1N···O2^i^	0.87 (1)	2.00 (1)	2.8634 (18)	173 (2)
C17—H17···O2^ii^	0.95	2.37	3.225 (3)	149
C38—H38*C*···O1^iii^	0.98	2.45	2.936 (3)	110

Symmetry codes: (i) *x*+3/2, *y*+3/2, *z*+1; (ii) *x*, −*y*+1, *z*+1/2; (iii) −*x*+3/2, −*y*+3/2, −*z*+1.
